# Suicidality in women nurses: A critical interpretive synthesis

**DOI:** 10.1016/j.ijnsa.2026.100516

**Published:** 2026-03-05

**Authors:** Anna Conolly, Hilary Causer, Jenny Oates, Cathy Shannon, Emily Knight, Chinenye Anetekhai, Ruth Riley

**Affiliations:** a School of Health Sciences, University of Surrey, Guildford, Surrey, GU2 7YH, United Kingdom; b South Eastern Health and Social Care Trust, Ulster Hospital, Upper Newtownards Rd, Dundonald, BT16 1RH, United Kingdom; c Nursing and Midwifry, University of Bolton, Deane Road, Bolton, BL3 5AB, United Kingdom; d Nursing and Midwifry, Birmingham City University, Birmingham, B4 7BD, United Kingdom

**Keywords:** Distress, critical approaches, feminism, literature review, nurses, suicide, suicidality, and women

## Abstract

**Background:**

The suicide rate among women nurses in the global north is considerably higher than the rate among male nurses and among women in other occupations. Research in this area is expanding but often appears similar because it is dominated by quantitative methodologies. This dominance is troublesome because quantitative approaches limit the questions asked, focusing on what is happening, rather than why it is happening. Individual-level explanations similarly dominate, sidelining both structural factors and lived experience. These limitations produce incomplete evidence that can lead to ineffective policy.

**Objective:**

To present a critical interpretive synthesis that aimed to identify how suicidality in women nurses is represented in research, the gaps within this representation, and the political, social, and personal consequences of these gaps. The occupational disparities women nurses face have been overlooked by previous researchers.

**Design:**

A critical interpretive synthesis approach was used with collaborative involvement from nurses who had relevant lived experience. These contributors participated throughout data extraction, interpretation, and synthesis to ensure that practical insight and real-world understanding informed the findings.

**Settings:**

Texts were included if they referred to nurses working in any form of health care setting across the globe.

**Participants:**

The review team consisted of seven members. Four were nurses with lived experience of suicidality who contributed contextual and analytic expertise.

**Methods:**

A total of 11,188 texts were screened. Reviewers completed full text assessments of 498 articles. Studies were organised by methodology, and a sampling frame was used to ensure balanced representation from a range of methodological traditions and discussion papers. Fifty studies were included in the final synthesis. Data extraction was guided by critical questions and an intersectionality checklist. A meta matrix was used to compare interpretations across studies.

**Results:**

Most studies used quantitative methods and presented suicidality as an individual problem associated with pathology or personal weakness. Recommendations commonly focused on screening or resilience training. Structural contributors, such as workload, staffing pressures, discrimination, psychological safety, and migration-related challenges, were often overlooked. Interactions between gender, ethnicity, and social position were rarely explored. Real life experience perspectives were under-represented.

**Conclusions:**

Individualisation, responsibilisation, and pathologisation obscure the root causes of distress and place the burden of blame on nurses. The result is poor quality and unrepresentative evidence that contributes to policies and practices that do not prevent suicide among women nurses.

Registration: osf.io g74uk, OSF Registries, Registered 01/07/2024

**What is already known**

• Women nurses have a higher risk of suicide than women in other occupations and male nurses in several high-income countries.

• Research on this topic is dominated by quantitative studies that focus on individual risk.

• Workplace conditions and wider social contexts are often overlooked in research.

Alt-text: Unlabelled box dummy alt text

**What this paper adds**

• We bring together diverse studies and lived experience to show how research commonly portrays suicidality in women nurses and where important gaps remain.

• We report that many papers emphasise individual pathology while downplaying staffing, workload, discrimination, migration, and other contextual pressures.

• We set out practical directions for future research and policy that prioritise safer, more supportive workplaces alongside access to mental health care.

Alt-text: Unlabelled box dummy alt text

## 1 Introduction

In the global north, the risk of suicide in women nurses is higher than male nurses and women in other occupations (Alderson et al., 2015; Davidson et al., 2021; Milner et al., 2016). In the United Kingdom (UK), the risk of suicide in women nurses is 23% higher than women in other occupations (Office of National Statistics, 2017). Globally, nursing is a female-dominated profession with a significant constituency from the global ethnic majority (Kurup et al., 2024). Despite this, most suicide literature and policies fail to account for the intersectional experiences of nurses (Causer et al., 2026). Many researchers use suicide data from death registers and other secondary data sources (e.g., Davis et al., 2021; Petrie et al., 2023) with quantitative, retrospective studies constituting the most evidence on suicidality in healthcare staff, including nurses. This research is based on individual risk factors, such as psychiatric illness, yet does not account for ethnicity or wider contexts, such as occupation, traumatic work events, or personal challenges.

Researchers who link suicidality to psychiatric diagnosis imply that the cause of suicide is individual pathology. Critical suicidologists, exemplified by Marsh’s (2010) foundational contribution, have problematised the insufficient critical analysis within suicidality research and exposed the weakness of its dominant assumptions (Rimke, 2016; Cohen, 2017). Bergey (2017) critiques the medicalisation of mental health due to its associations with medical imperialism, the social control of deviance, and the de-politicisation of social problems. This framing that positions suicide as the result of individual pathology and privileges medical, quantitative knowledge over other forms of knowledge can be viewed as an epistemic injustice (Fricker, 2007). Fricker’s (2007) definition refers to the wrong done to someone in their capacity as a knower by marginalising or dismissing their knowledge and experiences. Feminist researchers have highlighted that suicidal distress is rarely framed within the context of oppression and injustice (Cohen, 2017), overlooking political, economic, socio-cultural, and system-level factors that could lead to alternative interpretations of suicide in nurses(Fullagar & O'Brien, 2015). From our critical interpretive synthesis, we aimed to examine how suicidality in women nurses is constructed in existing research by identifying dominant discourses that have produced and maintained current knowledge(s) of suicidality in women nurses, paying close attention to alternative or excluded discourses. We aimed to map, situate, and critique the knowledges generated and maintained by researchers in this area and to examine their consequences. Guided by the question, “How is suicidality represented, what gaps exist, and with what consequences?”, we examined dominant and neglected discourses shaping knowledge, practice, policy, and research. We use *suicidality* to mean suicidal thoughts, behaviours, and deaths and *psychological distress* to denote emotional suffering without suicidal intent. We separated these terms deliberately, as they are often conflated in ways that obscure distinct contexts and contributors. We use *women nurses* to reflect the terminology and binary reporting of included studies. In line with contemporary usage, *woman/women* refers to gender identity and *female* to sex as reported in primary studies; however, most papers did not define or distinguish these terms, treating sex and gender as interchangeable and limiting analytic nuance.

During the last decade, there has been a significant increase in the awareness of mental health, with public health messaging stating that poor mental health could happen to anyone (Pescosolido et al., 2021; Cohen, 2017). Concurrently, neoliberal discourses continue to pathologise and individualise women’s problems through surveillance and self-regulation. Women are expected to fit in, to manage their personal and professional lives, and overcome *their* problems through constant self-improvement (Orgad and Gill, 2021). Interventions are employed to address psychological and suicidal distress in nursing through self-care and resilience discourses (Cooper et al., 2020). Resilience training and related messaging rely on individual-based interventions, and, thus, much resilience-based nursing work can be viewed as “psychocentric,” the individualization of human problems, through victim-blaming in a reductionist, determinist, positivist, and pathological individualist manner (Rimke, 2016). We view this approach as effectively restricting mental health narratives and obscuring wider organisational, social, political, colonial, and cultural contexts (Cohen, 2017).

Significant workforce challenges and legacy issues combine with an increasing workload to detrimentally affect the wellbeing of nursing staff (Maben and Conolly, 2024). Decades of workforce underfunding, sparce planning, and significant staffing shortages have led to notable structural challenges within nursing (Leary, 2017; Leary, 2019). Nurses disproportionately experience workplace violence, harassment, racism, bullying, and feeling psychologically unsafe that contribute to poor mental health, low job satisfaction, burnout, and retention problems (Jones et al., 2022; Maben and Conolly, 2024; Edmondson, 1999). This narrow framing of *the problem* of women nurse distress and suicidality limits our understanding of the contexts in which it occurs. In this review, we argue that a broader, holistic approach is necessary to clearly understand women nurse suicide. We identify how suicidality in women nurses is epistemically unjust due to its representation in research discourses, and we characterise the political, social, and personal consequences, explore the critical gaps, and incorporate stakeholder perspectives.

Nurses are keenly aware that the pressures of everyday practice, including heavy workloads, low staffing, limited support, and demanding work environments, have a real effect on their wellbeing. Yet when suicidality among women nurses is discussed, these wider pressures are often missing from the conversation. This synthesis brings together what is currently known to show why it is important to consider the realities of nursing work when trying to understand these experiences. It also points to the gaps in current knowledge and explains why clearer and more inductively-derived research is needed to support effective prevention efforts.

## 2 Methodology

Critical interpretive synthesis distinguishes itself from other types of review by emphasising theory development, critical orientation, and flexibility (Dixon-Woods et al., 2006). We adapted the critical interpretive synthesis by integrating Bacchi’s (1999) What’s the Problem Represented to Be framework to guide problematisation with Arribas-Ayllon and Walkerdine’s (2017) discourse analysis to interrogate subjectivity formation and intersectional sensitivity (as discussed in the analysis section). This hybrid approach allowed us to critically examine the content and silences within the literature. The product of the synthesis is not aggregations of data but theory grounded in the studies included in the review. We adhered to the procedures outlined in our preregistered Open Science Framework protocol (osf.io/g74uk). Critical interpretive synthesis questions hidden aspects of literature's agenda by including “absence or discursive silence”:

“…in developing a critical review, what is not said is as important as what is said. The CIS [critical interpretive synthesis] approach is interpretive in that lines of argument are developed through the integration of the literature with the authorial critique in a creative and inductive process” (Collier‐Sewell, 2023, p3).

Similar to Collier‐Sewell (2023) review, in this paper, our findings are integrated within the discussion. Depraetere et al. (2021) identified the key features researchers use when conducting a critical interpretive synthesis, and we refined these to form the methodology (Table 1). Our compass question was: how is nurse suicidality represented in current research, and what critical gaps exist in this representation and with what political, social, and personal consequences? The steps we took in this review are summarised in Table 1.

**Table 1:** Review steps.

**Step 1**	**Formulation and conduct of broad search strategy** • Involved electronic database search, reference chaining and expert consultation.
**Step 2**	**Inclusion and exclusion criteria setting** • Flexible inclusion criteria to select sources that are directly relevant to the research question and the emerging theoretical framework. Refined by AC, HC, RR, JO, CS, EK, CA.
**Step 3**	**Sampling** • Purposive sampling used to incorporate a mixture of research results including studies with varying methodologies as well as reviews and commentaries. Refined by AC, HC, RR, JO, CS, EK, CA.
**Step 4**	**Data extraction and interpretive synthesis** • The identification of themes was guided by a series of critical questions produced iteratively with (AC, HC, RR, JO, CS, EK, CA) • AC wrote critical summaries based on these questions. • Advisory group / study team extracted data on > 50% texts (AC, HC, RR, JO, CS, EK, CA) • Key concepts and interpretations were mapped into a “meta-matrix”. • Themes were generated by AC and reviewed by HC, RR, JO, CS, EK, CA. • A synthesizing argument was formulated by AC and reviewed by HC, RR, JO, CS, EK, CA. • AC drafted the paper. Drafts were reviewed by HC, RR, JO, CS, EK, CA

### 2.1 Searching and sampling strategy

To identify as many potentially relevant studies as possible, critical inpretative synthesis reviews employ a broad searching strategy (Depraetere et al., 2021). We conducted an extensive initial scoping review that helped to inform our search terms and inclusion/exclusion criteria. Our search terms are in Table 2.

**Table 2:** Keywords and databases.

Keywords	Nurs*
	suicid*
	Psych* distress*



Databases	PsycINFO (n = 3200)
	PubMed (n = 2800)
	Web of Science (n = 1989)
	CINAHL (n = 1635)
	Embase (n = 598)
	Scopus (n = 554)
	MEDLINE (n = 410)

We undertook further searches that included reference chaining and expert consultation. Our inclusion/exclusion criteria are shown in Table 3 below. Our broad criteria meant inclusion of a global literature of all nurse suicidality/distress papers. We uploaded the results into Covidence (an online tool for systematic reviews). AC screened results, with two co-authors (HC and RR), each screening 5% of all titles and abstracts. In the first stage of screening, we marked records as possible if they provided insight into the study’s compass question. Full-text copies of the remaining records were retrieved and uploaded to Covidence for final screening.

**Table 3:** Inclusion and exclusion criteria.

We selected items based on the following seven criteria:	
(1)	The literature was published between January 2013 and August 2024.
(2)	Studies were included that referred to registered nurses, excluding student nurses.
(3)	Due to restrictions in the authors’ language proficiency, information provided only in English was reviewed.
(4)	In terms of geographical relevance, published research from low- and middle-income countries (as defined by the World Bank) was included.
(5)	In terms of study characteristics, any methodology was to be included (e.g. quantitative; intervention studies; mixed methods studies; survey studies; qualitative studies; reviews/ commentaries/ discussions.).
(6)	Given our focus on nurse suicide or suicidality or psychological distress (including suicide or psychological distress during coronavirus disease), nurses' views towards patient suicide were excluded.
(7)	Papers were included that had a broad focus on the suicidality of either the general population or healthcare workers and then specifically discuss nurses in their findings. If the findings did not discuss nurses specifically, then the paper was excluded.

We included articles from low- and middle-income countries to reflect on homogeneity of literature from differing parts of the globe. We included literature on coronavirus disease as this represents a before and after moment in terms of the distress of the nursing workforce; our nurse advisory group wanted this subsection of literature to be included.

**Fig. 1:**
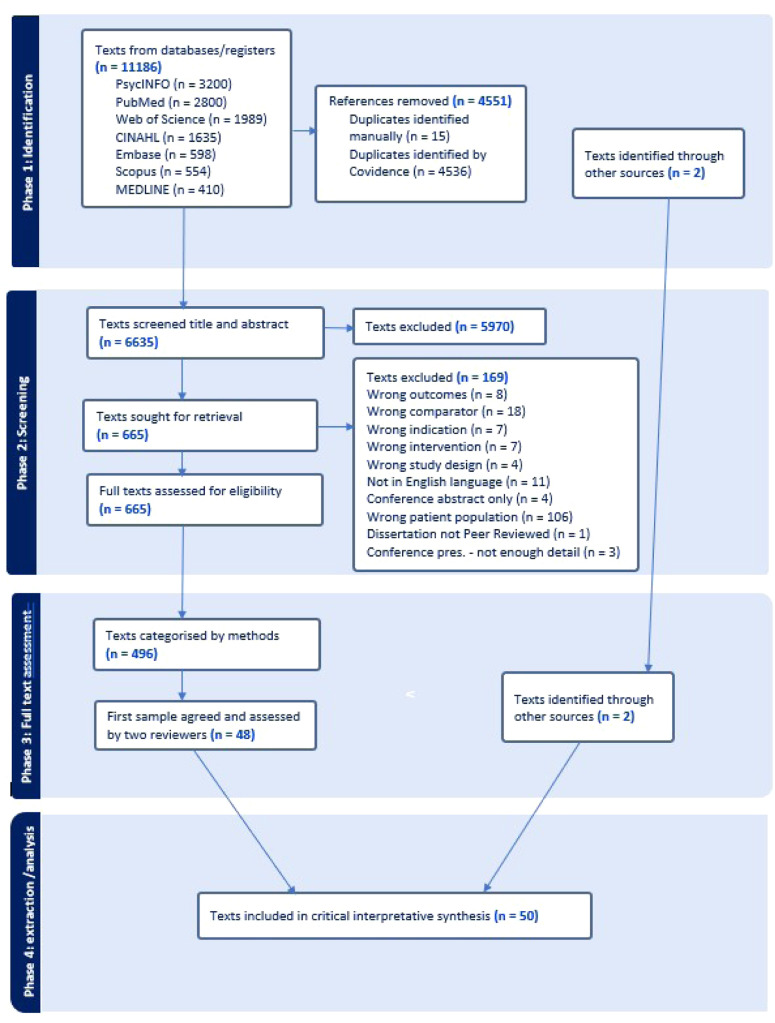
Literature search and study selection flow diagram.

Data analysis in critical interpretive syntheses is an iterative process involving detailed inspection of papers, identifying recurring themes, developing a critique. The selection of the literature was carried out in phases (Fig. 1). The first phase was a systematic search of electronic bibliographic databases. Phase two involved screening by title and abstract. Disagreements at each stage of screening were resolved through discussion between reviewers, with escalation to a third reviewer when consensus was not immediately reached.

Phase three was a full text review of 498 articles undertaken by three reviewers (AC, HC, RR). Each reviewer examined the records independently to assess inclusion; we discussed and resolved any discrepancies. At this point, we categorised the 498 texts that met the inclusion criteria by methodology and devised a sampling frame to ensure spread of papers. After selecting and screening the studies, we applied our analytic technique to the articles retrieved. As per other critical interpretive synthesis reviews, “high value” articles were included (Mattison et al., 2020, p4). “High-value” referred to texts offering conceptual, contextual, or discursive insight relevant to our compass question, those that illuminated problem representations, exposed silences, or critically-interrogated assumptions. Studies that repeated descriptive findings without adding interpretive depth were excluded.

### 2.2 Data extraction and interpretive synthesis

Phase four involved importing articles into Covidence for data extraction. The data extraction form was iteratively designed with the review team. To critically assess dominant discourses as institutionalized patterns of knowledge that govern the formation of subjectivity (Arribas-Ayllon and Walkerdine, 2017), we reflected on and incorporated their five-step approach to Foucauldian discourse analysis. These steps attend to the identification of discursive objects, subject positions, discursive practices, practices of power, and their effects. This was simplified through our adoption of Bacchi’s (1999) approach. The critical guiding questions that comprised our data extraction form were adapted from Arribas-Ayllon and Walkerdine’s (2017) and Bacchi’s (1999) approach. This aided our thinking around discourse constructs and the production of problems (Riemann, 2023). Critical interpretive synthesis does not employ conventional quality appraisal, so we used a credibility lens to consider transparency of methods, appropriateness of design, coherence between findings and claims, and reflexive awareness of researcher positioning.

To consider intersectional or overlapping identities, such as gender and ethnicity, we incorporated the Strengthening the Integration of Intersectionality Theory in Health Inequality Analysis checklist (Public Health Agency of Canada, 2022). Beyond noting its absence in primary studies, we applied intersectionality by examining how gender, ethnicity, migration status, and occupational conditions interacted in producing discursive silences across texts. AC wrote critical summaries of every text based on the data extraction form. In terms of the synthesis, we were inspired by Howes and Warwick’s (2023) description to “push beyond the original data to a fresh interpretation of the phenomenon under review” (Skewes McFerran et al., 2017 p67) by problematizing both the literature and its construction to better hear unspoken voices (Howes and Warwick, 2023). After reviewing the critical summaries and data extraction form for each text, we constructed a “meta-matrix” to present ideas together in tabular form for comparison and categorization (Miles and Huberman, 1994). The meta-matrix collated critical summaries and thematic codes across all included texts. Coding began deductively using categories derived from Bacchi’s questions and our intersectionality checklist. Inductive codes were developed through comparison within the meta-matrix, capturing patterns, contradictions, and silences across texts. The four final themes and the overarching model were agreed through iterative discussion, checking interpretive coherence and ensuring alignment with the compass question. This process ensured that multiple perspectives were considered and that the synthesis reflected a shared understanding of the data.

### 2.3 Review team and co-production

We are a multidisciplinary team comprising social scientists and academics with nursing backgrounds. We have an advisory group of diverse nurses with personal or professional experience of suicide and distress. The lead author worked collaboratively with the advisory group members to ensure their participation was meaningful to them and the research goals and their involvement conformed to INVOLVE standards (NIHR, 2018). The advisory members were financially compensated for their time according to national guidelines and had the opportunity to access free counselling to mitigate any negative impacts. The nurse advisors were involved in question formulation, data extraction form design, data extraction, data analysis, and writing. Their input was instrumental to the analysis, and, therefore, this paper is a truly collaborative work. Advisory members highlighted how several texts implicitly blamed nurses, which led us to identify the theme of responsibilisation. They also challenged initial readings that underplayed workplace racism, prompting closer attention to missing intersectional discourses. The advisory group helped to introduce checks and balances guarding against framing the analysis according to a single perspective (Dixon-Woods et al., 2006). We have used the GRIPP SF- 2 guidelines for reporting on advisory group involvement (Staniszewska et al., 2017); see Supplementary Material. Advisory co-researchers contributed to screening, data extraction, coding, and theme refinement. Their interpretations were weighted equally with academic reviewers. Disagreements were resolved through discussion; experiential and academic perspectives shaped the final analysis. Throughout the paper, we use contemporary, non-stigmatising terminology (e.g., “died by suicide”) aligned to current guidance on safe language.

### 2.4 Ethical considerations

Given the sensitive nature of the topic, ethical considerations were integral. While the review did not involve primary data collection, we recognised the potential for emotional distress among advisory group members. We provided access to counselling services, ensured informed consent for participation, and adhered to trauma-informed facilitation practices throughout the review process.

## 3 Results of the synthesis

In-line with methodological conventions particular to this type of review, descriptive results and interpretive analysis were integrated. Fifty texts were included in this review, including quantitative studies, intervention studies, mixed methods studies, qualitative studies, reviews, and commentaries/discussions/editorials. As outlined in Table 1, screening was completed by more than one person at almost every stage of the review. All texts had data extraction completed using the data extraction form with at least two people conducting data extraction on 28 texts representing >50% of the sample. Critical summaries were written by the lead author on every text. This process ensured extensive inter-rater reliability throughout the sample (Elliott, 2018). A full overview of characteristics of the included studies is provided in the Supplementary Material.

From our synthesis, we found that most research investigating suicidality employed discourses that attributed blame toward nurses for the elevated rates of suicide among women nurses in several ways: Discourses that imply or produce blame (Section 4.1 below), the legitimacy of claims (Section 4.2), minimised or missing discourses (Section 4.3), and positionality and emotion work (Section 4.4). The patterns described above summarise how suicidality is framed across the sample; below, we offer a critical interpretation of the implications of these discourses. (Dixon-Woods et al., 2006; Howes and Warwick, 2023). Similar to Howes and Warwick (2023) and Collier‐Sewell (2023), we found that embedded within most of the texts, research was being *conducted of, not for,* nurses. The problem, rather than being conceptualised as due to unfair working conditions and oppressive societal contexts, was positioned as being internalised in nurses. Discourses insufficiently interrogated the impact of oppressive and discriminatory practices pertinent to internationally-trained nurses from the global ethnic majority, resulting in missing or minimised discourses and unrepresentative knowledge that was colour-blind and ethnocentric.

**Fig. 2:**
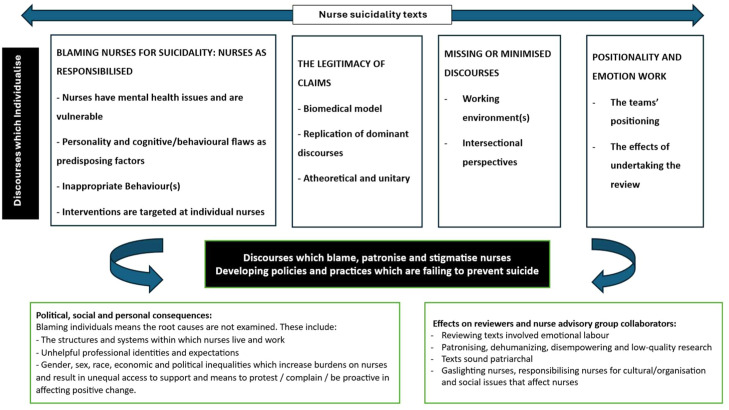
Synthesising model showing how dominant discourses in the literature construct suicidality in women nurses and the consequences of these constructions.

Discourses that attribute blame to nurses consequently minimise and silence diverse experiences and contexts, contributing to distress. Individualisation, responsibilisation, and pathologisation are the cause, while minimisation, institutional gaslighting, and silencing are the effects. The advisory group’s emotional reactions were essential in helping us name “institutional gaslighting,” as their reflections revealed how these discourses delegitimised nurses’ experiences and intensified responsibilisation. Consequentially, poor-quality and unrepresentative evidence is reproduced that leads to the development of policies and practices that fail to prevent suicide. Fig. 2 depicts the interpretive line of argument generated through the critical interpretive synthesis. At its centre are the dominant discourses identified across the sampled literature, including biomedical framing, individualised risk-factor models, responsibilisation narratives, and silence around organisational, intersectional, and structural conditions. Arrows show how these discursive patterns shape what is considered “the problem,” locating distress and suicidality within nurses’ personalities, behaviours, or presumed pathologies. The model illustrates how these discourses marginalise contextual explanations (e.g., unsafe staffing, discrimination, workload, migration pressures), reinforce epistemic injustice, and produce an evidence base that underrepresents the lived experiences of diverse women nurses. This, in turn, leads to policy and practice responses focused on individual screening, surveillance, and resilience training rather than addressing systemic or structural drivers. Overall, the figure conveys the central message of the synthesis: that current research discourses obscure associated factors, reproduce stigma and responsibilisation, and contribute to policies that are unlikely to reduce suicidality among women nurses. The Supplementary Material illustrates the texts associated with each theme.

## 4 Discussion of results

### 4.1 Discourses that imply or produce blame: Nurses as responsibilised

Many of the texts in the sample, particularly quantitative-oriented studies, attached blame to nurses for suicidality, rather than their working conditions, environmental, systemic, or social contexts that may contribute to nurses’ distressed emotional state. These discourses justify the adoption of suicide prevention interventions that focus solely on the individual, thus pathologising nurses. Interventions include mental health assessments and screening or improving access to support, rather than addressing the root causes of the problem. Blame was attributed in several different ways.

#### 4.1.1 Nurses have “mental health issues” and are vulnerable

Many texts referred to nurses who experience suicidality as having “mental health issues” (e.g., Barnes et al., 2023; Choflet et al., 2021; Wei, 2023) or struggling with “emotional issues” (Accardi et al., 2020). Nurses’ "emotional states" were analysed, with some texts characterising nurses as suffering from "intense affective states", including feelings of loneliness, hopelessness, desperation, and loss of control (Accardi et al., 2020). This was reported without consideration of environmental factors that may have caused these "emotional states". For instance, several survey researchers attributed suicidality to “emotional instability” or “pre-existing issues” without discussing workload, trauma exposure, or organisational pressures contributing to distress (e.g., Jahan et al., 2021; Li et al., 2024; Lascelles et al., 2023). The texts positioned nurses who had mental health issues or similar “vulnerabilities” (Lascelles et al., 2023, p2) as separate from those who did not. Scambler’s (2018, p767) work on “shame and blame” that draws on Goffman’s (1986) ideas concerning “othering” as a means of distinguishing what is “normal” in relation to the “abnormal” other through labelling resonates here. Accordingly, through the texts, nurses can be seen to have been *othered*, described overwhelmingly in terms of having deficits or a (biomedical) pathology that requires fixing or treatment. Suicidality was linked to “depression, burnout, anxiety, post-traumatic stress” (Melnyk et al., 2024,p.5) in many texts, with depression and burnout often mentioned as risk factors for nurse suicidality. Depression is often treated as an individual condition arising from internal pathology (Cohen, 2017). Burnout is more complex, and, while often seen within an occupational context, the individual is still pathologized as they are deemed to lack resilience or to have “maladaptive” coping strategies in response to work-related problems (Petit et al., 2024,p105).

The texts often characterised mental health conditions as existing *prior* to becoming a nurse. Examples include "pre-existing" mental health conditions, (Jahan et al., 2021, p1709) “cognitive frailty” (Li et al., 2024,p2), and "predisposing" factors (Davis et al., 2021, p652). Prevalent among quantitative research was the notion that nurses’ mental ill health could be attributed to childhood trauma, maltreatment, or adversities (Li et al., 2024; Jahan et al., 2021; James et al., 2023; ). Rebair et al. (2024) referred to nurses having “biological” problems that were not defined but indicated individual mental health problems (Cohen, 2017). Lascelles et al. (2023, p1) used the term "individual vulnerabilities including pre-existing mental health problems" to attribute causation of suicidality to women nurses.

Other texts frequently implied that nurses experience higher rates of mental illness and directly linked this to suicide, despite there being little evidence to support such claims (Marsh, 2010). Accessing psychiatric services prior to suicide was connected to nurses having mental health problems and blamed due to their “emotional problems” (Groves et al., 2023,p396). Emphasis was placed on a high rate of historical prevalence of mental illness among nursing professionals, as opposed to other professions, without substantiating these claims (e.g., Groves et al., 2023; Davidson et al., 2020). However, there is little evidence for this association, and researchers have now reported that one in four nurses, a rate similar to women in other occupations, were in contact with specialist mental health services in the year before death, (NCISH, 2020,p9).

#### 4.1.2 Personality and cognitive-behavioural flaws as predisposing factors

Attribution of blame was also evidenced in the texts by personality traits, such as perfectionism, or even displaying empathy, and cognitive-based behavioural attributes. For example, in Rebair et al. (2024, p37) report, a diagram was used to visually represent causes of suicidality, including issues that generated “vulnerability” in nurses, such as “biological, traumatic early experiences, personality traits, situational factors”. They state the risk for suicidal ideation is elevated by “perfectionism” (Rebair et al., 2024, p66). Personality traits were referred to in Alderson et al. ’s (2015,p97) review that stated "empathy is considered a central part of the health-care providers’ role but can also impact their mental health". Lange’s (2023,p29) commentary stated, “nurses who possess type-A personalities are self-critical” and “face a 20% increased risk of attempting suicide”. Personality traits and cognitive-based behaviours were used in some quantitative texts to categorise their sample and characterised suicidality as being internal to individual nurses. For example, Li et al. (2024, p7) categorised participants into the following groups: "the emotional problem group; lower emotional problem group; resilience and social support group; childhood trauma group". Thus, pathologising individuals and distracting from attempts to locate and alter systemic and social contexts that may have contributed to nurses distressed emotional state. Even in texts where personality traits were not the main focus, they were referred to in discussion sections. For example, Stelnicki et al. (2020,p232) cited traits of “high emotional attention”; e.g., “individuals who are highly emotionally perceptive, low self-esteem, and increased anxiety” as associated with “an increased risk for suicidal behaviours”. This blame and criticism of nurses’ empathy and perfectionist qualities appears contradictory because it runs counter to ideas of personality traits, such as compassion, being synonymous with the archetype of a “good nurse” (e.g., Hoeve et al., 2014). The identification of personality or cognitive traits obfuscates root or environmental causes of nurse distress and thus overlooks environment, system, or society-focused solutions to prevent distress before it occurs. Researchers have discounted personality traits as predicting mental distress due to evidence that traits can be taught through positive reinforcement and can, therefore, be viewed as skills that can be developed according to preference or reinforcement (Biglan and Hayes, 2016; Fryling, 2013).

#### 4.1.3 Inappropriate behaviour(s)

Many of the texts characterise nurses as having causative/contributory "health behaviours" (Groves et al., 2023, p397) that position nurses as to blame for suicidality. Often written in a paternalistic and infantilising manner, as if nurses are incapable of making good (or correct) choices, the texts highlight an array of problematic behaviours, including nurses avoiding “self-care”. For Hofstetter and Mayer (2023, p31), "nurses frequently prioritize patient care over self-care”, and they argue that nurses’ fear of being "devalued, dismissed, and dehumanized" means that they often work when they are not able to (known as “presenteeism”). Researchers have shown that nurses’ feelings of stigma within healthcare settings are often attributable to feeling psychologically unsafe within workplaces due to poor workplace cultures (Maben et al., 2023; Laker, 2019). Some texts individualise nurses’ refusal to seek help due to stigma by emphasising the fear nurses feel (Hofstetter and Mayer, 2023; Kelsey et al., 2021), as if this is something that manifests unreasonably within the individual. In a similar manner, Eder and Meyer (2022) reported that nurses "self-endanger" by self-sacrificing “themselves for their work”. Within these discourses exists a damning moralisation of nurses that attempts to circumscribe their behaviour by subjecting them to judgemental surveillance. For example, some texts implied nurses “should” engage in better self-care or resilience, even when the same researchers acknowledged understaffing, unsafe cultures, and punitive managerial responses (e.g., Berry, 2021; Kelsey et al., 2021; Wei, 2023).

A further health behaviour is the notion that nurses “misuse substances” or have a "compulsive use of substances" (Barnes et al., 2023,p.10) that leads to their suicidality. In some of the texts reviewed (e.g., Davidson et al., 2021), there is evidence provided that nurses die by suicide using legal and illegal drugs, alcohol, and medications; however, there is minimal evidence provided within the texts that nurses undertake prolonged substance use. In some texts (e.g., Choflet et al., 2022; Groves et al., 2023), the length of author reflection on the use of substances is overproportionate to the significance of their findings. These texts fail to examine wider social or work-based contexts that might have produced this behaviour. The over-focus on method of suicide distracts from the contexts contributing to suicidality.

#### 4.1.4 Interventions are targeted at individual nurses

The attribution of blame to nurses distracts and detracts from a combination of systemic and social contexts, thus providing the rationale for researchers to focus on those who already show signs of distress, as opposed to preventing the root causes of distress. The Healer Education Assessment and Referral programme is the most widely advocated for intervention. This programme was originally developed as a physician-suicide prevention programme but was expanded to nurses. It involves a 4-phase intervention consisting of education, mental health screening, assessing, and referring nurses at risk of suicide (Sofer, 2021). Eighteen of the texts (including Accardi et al., 2020; Sofer, 2021; Barnes et al., 2023; Basu et al., 2023) advocate for the Healer programme without citing supporting evidence. For example, Sofer (2021, p14) refers to it as "signs of progress**"** and states it is “endorsed by the American Medical Association as a best practice in suicide prevention”; Lee et al (2021, p4) mention the ability of programmes, such as the Healer, “to lower the stigma of seeking help, [and offer] proactive anonymous screening to identify nurses at risk, debriefings after stressful incidents, and counselling and connections to treatment”.

A mental health screening tool as an intervention implies the role of depression in suicidality that is not strongly substantiated (Marsh, 2010). Screening individual nurses ignores the environmental and systemic issues that may contribute to nurse suicidality. Like the Healer programme, a proposed intervention mentioned by two of the texts is MINDBODYSTRONG© (Melnyk et al., 2024; Lange, 2023). This cognitive-behavioural skills building programme aims to help nurses develop coping skills to decrease depressive symptoms and anxiety (Lange, 2023). This programme has been endorsed by some of the research team behind the Healer programme, who have submitted a protocol for an intervention study to “determine the effects of mISP [Modified Interactive Screening Program] combined with the digitized MINDBODYSTRONG© versus the mISP alone on depression, suicidal ideation, burnout, anxiety, posttraumatic stress, healthy lifestyle beliefs, healthy lifestyle behaviors, and job satisfaction in U.S. [United States] nurses” (Melnyk et al., 2024, p3). If deemed to be successful, the MINDBODYSTRONG may supersede the Healer programme as the go-to recommended intervention for nurses showing signs of distress.

Other screening interventions targeted at individual nurses also appear in the sampled texts. These are seen as mandatory for nurses who experience certain risk factors. For example, nurses with “chronic pain” being screened for depression and “risky substance use” as part of their mandated wellness plan (Barnes et al., 2023); "sleep screening" to identify nurses at risk of sleeping less than 6 hours per night, with a view to prescribing cognitive behavioural therapy (Mao et al., 2024,p287). Resilience, a highly contested term in nursing workforce research (see Traynor, 2017; 2018; Conolly et al., 2022), is often mentioned in the sampled texts as an area for development (Wei, 2023; Berry, 2021). Li et al. (2024, p9) argue that “enhancing […] resilience stands out as essential for nurse staff exhibiting higher levels of suicidal ideation and non-suicidal self-injury" and " fostering resilience [is a] key component in comprehensive strategies to prevent and manage suicidal behaviours among nurses”. Berry (2021) argued during the pandemic that nurses should “increase our resiliency strategies”. Other areas for nurse improvement are mentioned; "programmes” that “focus on nurses’ special job motivation and attitudes to reduce self-sacrificing within their work" (Eder and Meyer, 2022, p46). Forceful language is often used to tell nurses how to improve; e.g., James et al. (2023, p104) argue “a systems level approach to *hardwire* coping strategies" needs to be used. In many of the sampled texts, the proposed interventions do not align with the data reported in the authors’ findings sections; quality and robustness are discussed below.

### 4.2 The legitimacy of claims

#### 4.2.1 Biomedical model

Most of the texts sampled utilised a similar reliance on the biomedical model and embedded objective, positivist approaches derived from Western medical science (Marsh, 2010). This shaping of knowledge brings a similar rhetoric to the texts premised on the notion that nurse suicidal behaviour can be predicted by demographic factors, such as gender, age, ethnicity, sexuality, and socio-economic status. These factors are interpreted as “risk factors”, and it is these, along with “predictors”, “warning signs”, and “protective or supportive factors” that dominate the discourse of nurse suicidality (e.g., Davidson et al., 2021; Ding et al., 2024; Petit et al., 2024). Other dominant elements of the biomedical model included validated measures/instruments that functioned to provide the illusion of scientific authority to authors’ hypotheses. However, significant flaws existed within these texts, even within research articles that had been peer reviewed and accepted for publication in high-ranking journals.

Texts often attempted to *overclaim* in their research, enabled by the authors’ use of biological and psychological terms to provide a scientific basis for claims that nurse suicidality was a pathology within. There is an increasing awareness that body and mind should not be treated separately (e.g. Koban et al., 2021); however, the evidence presented in the texts for treating them together was often flimsy. For example, Barnes et al. (2023,p10) biomedical/ physiological science discourses-based text involved a description of suicide “vulnerability” in nurses as resulting from:

“shared neural pathways, neuroinflammation, and feelings of hopelessness and burden are the bridges that entangle the diseases with each other".

The authors’ explanation of these terms and their association to nurse suicidality were not rigorously referenced, which raises questions regarding the scientific basis behind these claims. Barnes et al. (2023) text indicates the priority placed on examining pathologies within nurses, as opposed to looking at environmental and systemic reasons for suicidality.

This privileging of biomedical approaches is not unusual in nursing and medical research that has historically relied on public health surveillance using epidemiology and the monitoring of populations through the production of big data, often via governmental-based health population-wide surveys (Fullagar and O'Brien, 2015). This kind of data is still placed at the top of hierarchies of evidence in nursing and medical research (Mackey and Bassendowski, 2017). The objectification of suicidal people in mortality statistics has been the source of sustained debate within critical suicide studies (Hjelmeland and Knizek, 2010). Other critical theorists have found that in nursing research even qualitative work is often conducted through a positivist lens (Collier‐Sewell, 2023).

#### 4.2.2 Replication of dominant discourses

Discourses were found to be repeated, uncritically, from text to text within the sample. These repeated discourses often appeared in the discussion sections of texts and did not always follow on from research discussed in the findings sections. This is most apparent in article recommendations to reduce suicidality, with the repetition of one or two individualising interventions when the findings occasionally highlighted wider environmental contexts. This replication of discourse and recommendations, with the associated lack of criticality and originality, can be viewed as epistemological privileging and leaves the root causes of nurse suicide with insufficient interrogation. There appears to be a lack of curiosity among authors of the texts regarding knowing and understanding the lived experiences and perspectives of diverse women nurses.

#### 4.2.3 Atheoretical and unitary

When theory was used in the texts, it tended to be individualising. Theories included: job demands–resources model (Alderson et al., 2015); job demands-control (Whybrow et al., 2024); ideation-to-action framework and three-step theory (Basu et al., 2023); stress-diathesis theory (Ding et al., 2024; Rebair et al., 2024); strain theory (Wang et al., 2024); and interpersonal theory of suicide (Zohn and Hovis, 2024; Damirchi et al., 2019). The use of these theories enabled the othering and responsibilisation of nurses who were perceived to have internal problems or inappropriate behaviour. For example, in Davidson et al.’s research (2021, p28), the conceptual framework for the study was “the Interacting Risk and Protective model for understanding suicide utilized by the American Foundation of Suicide Prevention”. This model is premised on longer term and short-term biological, psychological, and social and environmental contributors to suicide risk, such as mental health conditions, physical health conditions, early life stress, history of abuse or neglect, family history, and history of head trauma, that are argued to “converge in the context of stressors” such as divorce, marital strain, job problems, pain, illness, or financial difficulties to increase risk for suicide (Davidson et al., 2021, p28). For Davidson et al. (2021, p29), “suicide risk fluctuates based on the interrelationship among risk and protective factors and stress”. The narrow use of theories that individualise women nurse suicide can be seen to result in discourses of pathologisation and blame.

### 4.3 Minimised or missing discourses

#### 4.3.1 Working environment

Many of the texts referred to organisational problems only once with no elaboration(e.g., Accardi et al., 2020). This minimisation is carried out to such an extent that work environment is rendered invisible, with nurses treated as an incidental population for research in some texts. For example, in Li et al.’s (2024, p2-4) cross-sectional survey, nursing as a profession is mentioned only in the introduction. The authors concentrate on psychosocial factors that they define narrowly as “cognitive frailty”, “childhood maltreatment”, “loneliness”, and “sleep issues”. These are issues that can be viewed as individual as opposed to environmental. This definition of psychosocial can be viewed as unrealistically narrow, as within broader literature, psychosocial is often defined as examining “the relation between intrapersonal psychological and environmental aspects” (Vizzotto et al., 2013,p 1578). In health workforce wellbeing research, greater scrutiny of occupational contexts is usually provided, including working environments, conditions, and cultures. The omission or lack of consideration of workplace contexts in the texts sampled is notable, particularly when there is substantive evidence to suggest that these contexts play a contributory role in emotional and psychological distress. This effective dismissal of the consequences of work-based environmental stressors and conditions is commonplace within the texts.

Some of the texts set out to examine one specific element of healthcare working environment, and this hyper-focus on one specific area seemed to give the authors permission to ignore wider workplace stressors. For example, in their consideration of violence in the workplace and how this affects nurses’ suicidal ideation, Ding et al. (2024) do not refer to any work-based, environmental, or social factors outside of workplace violence. Similarly, many of the post-2020 texts attribute poor, traumatic, and stressful working conditions as causative of nurse suicidality to coronavirus disease, as if these factors are solely attributable to the pandemic and did not exist previously. Texts such as Sofer (2021), Zohn and Hovis (2024), and Downs et al. (2024) reflect on the specific set of working conditions that the pandemic brought. Sofer’s (2021) discussion piece argues that coronavirus disease has made things tougher for women nurses in most aspects of their work, but this attribution of tough working environments as being caused by the pandemic renders invisible the poor and stressful working conditions that pre-existed or continue post-pandemic.

#### 4.3.2 Intersectional perspectives and personal, social and political consequences

The notion of social justice is often highlighted as a cornerstone of nursing due to nurses’ responsibilities for providing equitable and fair care for people from all backgrounds (Abu and Moorley, 2023). Likewise is the recognition of the social determinants that impact physical and mental health (Marmot et al., 2020). In their training, nurses routinely learn of the high-levels of health disparities, caused by unfair socioeconomic conditions, power dynamics, and poverty created by discriminatory systems and that contribute to inequalities in health (Abu and Moorley, 2023). It is striking that even with this awareness of the social determinants of health embedded in nursing, social justice approaches to suicide were notably absent from the texts sampled. Despite sustained evidence that women nurses are more likely to die by suicide compared to their male peers, gender and sex were rarely discussed. Although nursing can be traditionally viewed as a female profession and is also increasingly ethnically-diverse, the majority of the texts reviewed were gender and colour blind, with minimal attention paid to other intersectional identities (such as social class). For example, in Davis et al. (2021), although a major finding of their research was that women nurses were more likely to die by suicide, gender received no comment in the discussion of their research. This absence of discussions of gender was evident in quantitative work conducted globally, with examples from the US (James et al., 2023) to Bangladesh (Ding et al., 2024). When authors did refer to gender, there was an absence of critical interrogation. For example, Jahan et al. (2021, p1709) research in Bangladesh found that more women nurses are likely to have died by suicide, “which should be noted by employers”, but this was the authors’ only comment on gender. In the UK, a NCISH (2020) report gave more consideration to gender but did not critically interrogate gender-based oppression or wider contexts impacting women nurses. Milner et al.’s (2016,p.264) examination of mortality statistics gave consideration to the wider contexts that may contribute to nurse suicidality, as the authors stated that: “female professionals may still feel pressure to undertake child-care and household roles, leading to considerable gender role stress”. Nevertheless, overall, the structures and systems within which women nurses live and work were not considered. None of the included studies examined the impact of low pay, precarity, debt, or gender-based wage disparities on nurses’ distress or suicidality, despite clear evidence that women nurses often experience economic disadvantage within healthcare systems. Further, none of the included studies examined how government decisions, such as funding cuts, regulatory pressures, or staffing mandates, shape working conditions or contribute to distress and suicidality among women nurses.

This invisibility was replicated in identities related to race and ethnicity. In some texts, data had been collected on ethnicity and race but then not discussed, (Choflet et al., 2022; Davidson et al., 2021). The NCISH (2020) report features no reference to intersectional perspectives, differences of sexuality, nor analysis of ethnicity/race. There was one notable exception that tugged at this veil of invisibility: the US based discussion piece written by Wells (2024,p7), who advocated for the application of an “Equity Lens” to help understand, arguing that "it is essential to recognize that all nurses do not face the same workplace stressors”.

A notable absence in the texts was consideration of the impacts and consequences of nurses’ work-based migration. Many developed countries experience nurse shortages utilise overseas recruitment; e.g., in the UK between April 2021 and March 2022, a total of 23,444 internationally trained nurses joined the Nurses and Midwives Council register (NMC, 2022, p4). The pressures on nurses who leave their social networks behind to work in a new country whilst navigating unknown systems and work-place based discrimination (NHS, 2025) are currently invisible in the nurse suicidality texts. Internationally-educated nurses often work in under-resourced settings, encounter racism and exploitation, and face separation from support networks. These factors likely intersect with distress, though largely unexamined in existing studies.

The rendering invisible of intersectional perspectives serves to erase women’s identities and experiences from the narrative and belittles and minimises women nurses’ distress and suicidality. The texts did not attend to gender, sex, race, economic, caring, family, social, colonial, and political inequalities that place increased burdens on women nurses and provide them with unequal access to support and the means to be pro-active in affecting positive change through offering resistance or calling out inequality. Critical suicidologists have highlighted the importance of injustice, inequality, exclusion, and oppression (Marsh, 2010) when considering suicidality. This aligns with Patrica Hill Collin’s (2022) work, which posits that distress is framed by the powerful to sit within the individual not the system. Indeed, Spates (2015), in her work on African-American women, reminds readers that suicidality is a social justice issue, not an individual one. The reproduction of poor-quality and unrepresentative evidence leads to the development of policies and practices that enable the targeting of individuals through mental health screening and thus fail to prevent suicide. An alternative approach that holistically considers the contexts in which suicidality is produced would be more likely to recommend changes to the institutions and societal contexts that may be contributing to suicidality.

### 4.4 Positionality and emotion work

#### 4.4.1 The teams’ positioning

A core criticism of many of the texts sampled is that very little consideration was given to discussions of the authors’ positions in the research process. Our nurse reviewers felt curious about the identity and profession of the authors, as texts were often written in a style that distanced the authors from individual nurses. Affective or emotion work is frequently taken into consideration in both feminist research and in critical interpretive syntheses; e.g., the emotions of reviewers (Skewes McFerran et al., 2017), with spaces being created that accounted for reviewers’ reactions to the texts they were reviewing (Howes and Warwick, 2023). This is also important in both suicidality (Chandler, 2020) and feminist research.

#### 4.4.2 The effects of undertaking the review

Reviewing these texts brought up many emotions for our review team, requiring emotion work to process and manage these. For example, review members referred to the anger they felt “due to patronising, dehumanizing, disempowering messaging in the narratives and low-quality research”. Nurse reviewers commented that the “texts sound patriarchal” and that the literature could be viewed to be “gaslighting nurses, responsibilising nurses for cultural/organisation and social issues that affect nurses”. One nurse reviewer commented that:

*“[Reviewing the texts] made me feel quite cross and responsibilised. We are all encouraged to take responsibility for our own health, but these articles crossed the line. I disagree with comments about perfectionism being a fault when you are working in life support* (Nurse Advisory Group Member, 2025)*”*

These reflections directly informed our identification of “institutional gaslighting,” as advisors’ emotional responses illuminated how pathologising discourses minimise structural harm. In attending to emotion work within the review team, we adopted non-stigmatising language to avoid reproducing harm in our own framing.

Studies utilising registered death data were treated cautiously by the nurses, with one commenting that “the epidemiological data is used as a jumping off point to discuss depression; however, they do not have any clear data to evidence this”, and “there are multiple reasons why medical examiner data on death might not be a robust account of deaths by suicide”. Thus, the privileging of medicalised knowledge ultimately renders nurses’ experiences and wider contexts contributing to suicidality invisible.

## 5 Limitations

This review is subject to several limitations. The inclusion of only English-language texts may have excluded relevant studies from non-English-speaking contexts, potentially limiting the global applicability of findings. While the co-production approach enriched the analysis, the advisory group’s composition may not fully represent the diversity of the global nursing workforce. The integration of multiple critical frameworks, while methodologically innovative, may have introduced complexity that could affect reproducibility. The emotional labour involved in reviewing distressing content may have influenced the interpretation of findings, despite efforts to maintain reflexivity and transparency. Primary studies rarely defined or differentiated sex and gender categories. This restricted our ability to apply more precise, inclusive terminology within the synthesis. Sex/gender was reported in binary terms, limiting our ability to examine suicidality beyond binary categories. The absence of data on trans and gender-diverse nurses reflects a structural gap in the literature rather than a limitation of our review.

## 6 Conclusion

In this review, we have assessed and critiqued the dominant discourses, gaps, and (in)visibilities in nurse suicidality texts to examine the political, social, and personal consequences and to challenge the reproduction of poor-quality and unrepresentative evidence that informs policies and practices that fail to prevent suicide in women nurses. We found epistemological privileging of quantitative methodologies, an over-focus on individual risk factors, and over-reliance of the biomedical model has shaped knowledge on suicidality in women nurses. This has led to a pronounced epistemic injustice with diverse women nurses’ voices and experiences remaining unrepresented in suicide knowledge, while pertinent and potentially relevant contexts continue to be under-explored. The victim-blaming inherent in the pathologisation and individualisation of nurses has likely compounded the associated shame and stigma associated with suicidal behaviour. The privileging of knowledge, or epistemic injustice, reinforces longstanding discourses that continue to frame suicide as an individual problem: one to be fixed through the subsequent diagnosis and treatment of mental illness. Nurse researchers involved in this review actively resisted the power relations reproduced by medicalised discourses by highlighting the effect of oppression and injustices experienced by nurses as women, including the gendered-devaluing of nursing work.

Globally, there is an estimated shortfall of 13 million nurses worldwide (International Council of Nurses, 2023); recruitment policies in the Global North have resulted in the relocation of nurses from countries in the Global South. The wider contexts in which nurses work were overlooked in the texts we reviewed, with sparse attention paid to systemic issues like working in chronically-underfunded healthcare systems and oppression, including harassment, racism, bullying, and feeling psychologically unsafe due to poor workplace cultures. The UK policy landscape on this topic is examined in detail in Causer et al. (2026). Although the timely provision of effective mental health support is important, policy makers should avoid sticking-plaster approaches and becoming complicit in pathologising nurses. Employers and policy makers can deploy interventions that may meaningfully address the contributing factors of the problem with system-level solutions. Examples include safe-staffing legislation, anti-racism and psychological-safety initiatives, protections for internationally-educated nurses, and reforms to occupational health systems that shift from surveillance and risk-screening to supportive, non-punitive organisational responses.

We present this review as a call to action to researchers to use broad and flexible research methodology in research design. Future researchers should apply co-produced, participatory designs, qualitative longitudinal studies, or critical ethnographies capable of examining structural conditions and lived experiences over time. We encourage use of methods that will problematise and interrogate the environmental or context-driven nature of suicidality to more meaningfully-inform suicide prevention policies. We utilised authentic co-production with nurses to avoid further epistemic injustice. We have problematised academic texts with their supposed rigour and robust use of appropriate research design and methodology. The positivist models that we found to be dominant across the included texts did not consider the consequences of discourses that pathologise and stigmatise nurses. We found that diverse women nurses’ voices, experiences and references to unequal power dynamics, oppression, and injustice were invisible. As feminist researchers, we are reminded that the goal for feminist work is transformative action based on social justice that improves the lives of women. Thus, throughout this review, we worked with nurses, for nurses, in-line with feminist principles that consider wider systemic, social, political, and environmental contexts in which suicidality occurs to more reliably reduce distress and possibly prevent nurse suicide.

## Funding

This work was funded by the Wellcome Trust grant number 227397/Z/23/Z.

## Rights retention statement

This research was funded in whole, or in part, Wellcome Trust grant number 227397/Z/23/Z. For the purpose of Open Access, the author has applied a Creative Commons Attribution (CC BY) public copyright licence to any Author Accepted Manuscript version arising from this submission.

## CRediT authorship contribution statement

**Anna Conolly:** Writing – review & editing, Writing – original draft, Visualization, Validation, Supervision, Software, Resources, Project administration, Methodology, Investigation, Formal analysis, Data curation, Conceptualization. **Hilary Causer:** Writing – review & editing, Validation, Supervision, Methodology, Investigation. **Jenny Oates:** Writing – review & editing, Validation, Investigation, Formal analysis. **Cathy Shannon:** Writing – review & editing, Investigation, Formal analysis. **Emily Knight:** Writing – review & editing, Investigation, Formal analysis. **Chinenye Anetekhai:** Writing – review & editing, Validation, Investigation, Conceptualization. **Ruth Riley:** Writing – review & editing, Validation, Supervision, Methodology, Investigation, Funding acquisition, Formal analysis, Data curation, Conceptualization.

## Declaration of competing interest

The authors declare that no competing interests exit.

## Appendix Supplementary materials

Image, application 1
