# Supplementary material for: Suicidality in women nurses: A critical interpretive synthesis
Source: Int J Nurs Stud Adv. 2026 Mar 5;10:100516. doi: 10.1016/j.ijnsa.2026.100516 (PMC12997203; doi:10.1016/j.ijnsa.2026.100516)
Supplement: Supplementary file 1 [file mmc1.docx]

**Supplementary data**

**Table 4: GRIPP SF co-production aims and activities**

| **The aim of Involvement in the study** | **Examples** |
| --- | --- |
| Provide a clear description of the methods used for advisory group involvement in the study | Our proposal for the critical synthesis was presented to our advisory members prior to the funding application and commencement of the review. Advisory members gave input on our review objectives, review questions, search strategy and data extraction proforma. Advisory members were invited to join the review team, of whom three joined. They received training and support in critical synthesis methodology. They undertook review data analysis. The wider advisory group was invited to review and comment on initial findings presented by the team. The advisory collaborators contributed to the writing of the review paper. |
| Process—Report the results of advisory group involvement in the study, including both positive and negative outcomes | We refined our review questions to incorporate advisory perspectives. Advisory members had limited experience of undertaking reviews so we supported them with training on the techniques used. |
| Outcomes—Comment on the extent to which Advisory group influenced the study | This collaboration was central to our critical review, assuring us that our analysis was credible and reflective of nurses’ perspectives. |
| Comment critically on the study, reflecting on the things that went well and those that did not, so others can learn from this experience | There is a lack of co-produced research with nurses on their mental health and in suicide research. Our approach shows that nurses with relevant lived experience can actively be involved in the review and data analysis. Our advisory members are all busy professionals. Their contribution to this project, whilst paid, was in addition to their daily work requiring researchers to hold meetings in the evenings. Providing flexibility ensured accessibility and ensuring contributors were valued was key. This should be factored into future research. We paid advisory members £30 per hour for their contribution. We offered additional training and support to enable contributors to apply the methodology. Future researchers should factor this into costings for advisory group involvement. |

**Figure 3: Texts categorised by methodology**

**Table 5: Themes with exemplifying texts**

**
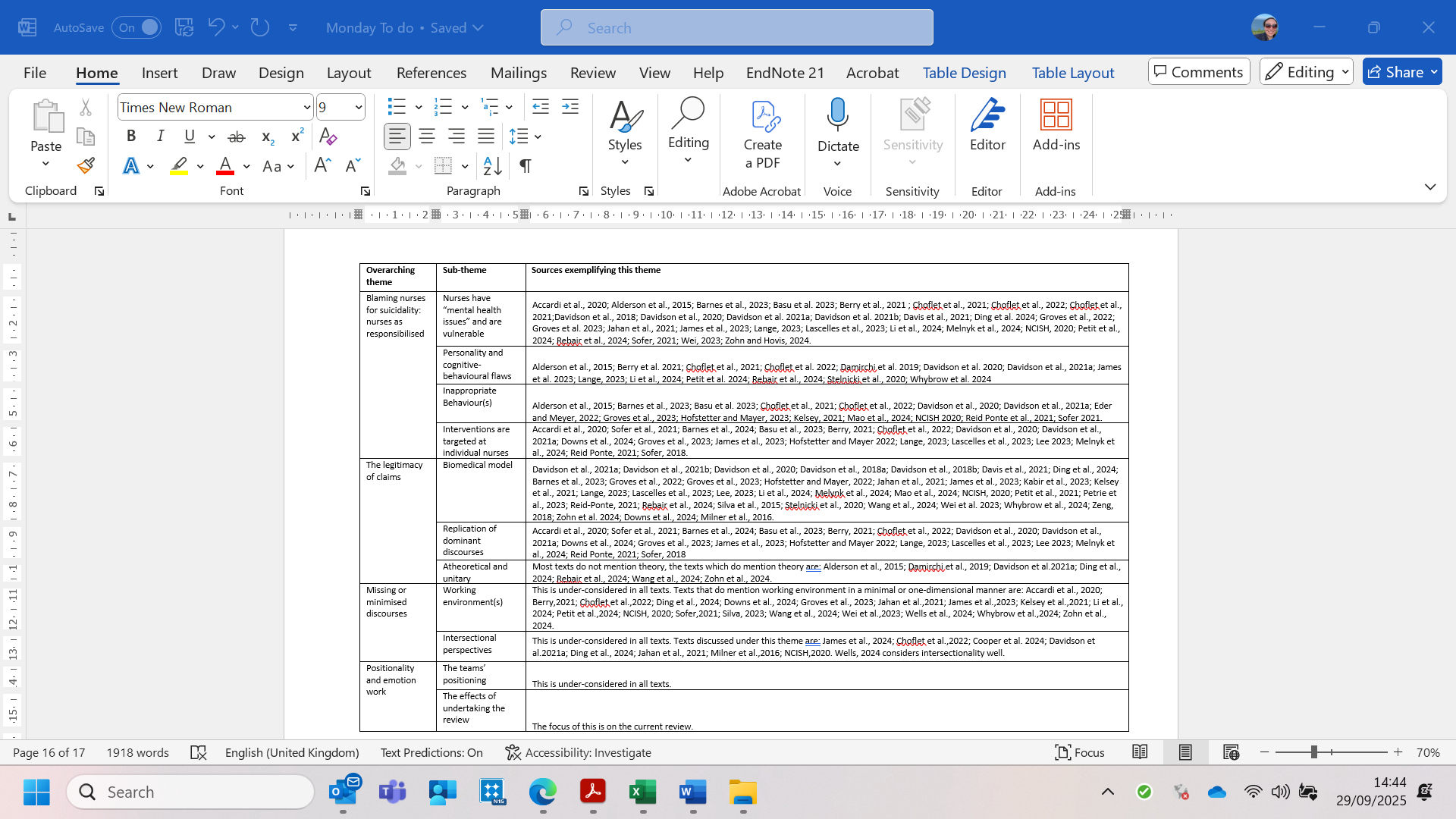
**

**Table 6: Description of the included studies**

| **Author and year** | **Title** | **Journal** | **No. of Citations**  **(google scholar on 04.06.25)** | **Country** | **Topic**  **(Distress/ completion/ ideation/ intervention)** | **Broad data category** |
| --- | --- | --- | --- | --- | --- | --- |
| Accardi et al. 2020 | Sustainability and outcomes of a suicide prevention program for nurses | Worldviews on Evidence-Based Nursing | 72 | United States | Intervention | Intervention study |
| Alderson et al. 2015 | Critical Review on Suicide Among Nurses What About Work-Related Factors? | Crisis-The Journal of Crisis Intervention And Suicide Prevention | 99 | United Kingdom | Completion | Review |
| Barnes et al. 2023 | Entangled: A mixed method analysis of nurses with mental health problems who die by suicide | Nursing Inquiry | 13 | United States | Completion | Mixed method |
| Basu et al. 2023 | Understanding nurse suicide using an ideation-to-action framework: An integrative review | Journal of Advanced Nursing | 8 | United States | Ideation/ completion | Review |
| Berry et al. 2021 | Combatting Nurse Suicide | Professional Case Management | 5 | United States | Intervention | Discussion pieces/commentaries/ editorials |
| Choflet 2022 | The nurse leader's role in nurse substance use, mental health, and suicide in a peripandemic world | Nursing Administration Quarterly | 18 | United States | Intervention | Discussion pieces/commentaries/ editorials |
| Choflet et al. 2021 | A comparative analysis of the substance use and mental health characteristics of nurses who complete suicide | Journal of Clinical Nursing | 36 | United States | Completion | Quantitative |
| Cooper et al. 2024 | Exploring work-related stressors experienced by mental health nurses: A qualitative descriptive study | Journal of Psychiatric and Mental Health Nursing | 5 | Australia | Distress | Qualitative |
| Damirchi et al. 2019 | The role of thwarted belongingness, perceived burdensomeness, self-efficacy and ego strength in predicting suicidal ideation of nurses | Health in Emergencies & Disasters Quarterly | 20 | Iran | Ideation | Quantitative |
| Davidson et al. 2020 | A Longitudinal Analysis of Nurse Suicide in the United States (2005–2016) With Recommendations for Action | Worldviews on Evidence-Based Nursing | 186 | United States | Completion/ intervention | Quantitative |
| Davidson et al. 2019 | Nurse suicide in the United States: Analysis of the Center for Disease Control 2014 National Violent Death Reporting System dataset | Archives of Psychiatric Nursing | 128 | United States | Completion | Quantitative |
| Davidson et al. 2018 | Testing a Strategy to Identify Incidence of Nurse Suicide in the United States | Journal of Nursing Administration | 41 | United States | Completion | Intervention |
| Davidson et al. 2021 | Exploring nurse suicide by firearms: A mixed-method longitudinal (2003-2017) analysis of death investigations | Nursing Forum | 16 | United States | Completion | Quantitative |
| Davidson et al. 2021 | Job-Related Problems Prior to Nurse Suicide, 2003-2017: A Mixed Methods Analysis Using Natural Language Processing and Thematic Analysis | Journal of Nursing Regulation | 62 | United States | Completion | Mixed method |
| Davidson et al. 2018 | Suicide Prevention: A Healer Education and Referral Program for Nurses | Journal of Nursing Administration | 88 | United States | Intervention | Intervention |
| Davis et al. 2021 | Association of United States Nurse and Physician Occupation With Risk of Suicide | Jama Psychiatry | 172 | United States | Completion | Quantitative |
| Ding et al. 2024 | Pathways linking workplace violence and suicidal ideation/non-suicidal self-injury among nurse staff: the mediating role of loneliness and depressive symptoms | BMC Nursing | 3 | China | Ideation | Quantitative |
| Downs et al. 2024 | Learning from Health Care Counselors’ Perspectives on Health Care Worker Distress: A Qualitative Analysis | HCA Healthcare Journal Of Medicine | 0 | United States | Ideation | Qualitative |
| Eder and Meyer 2022 | Self-endangering: A qualitative study on psychological mechanisms underlying nurses' burnout in long-term care | International Journal of Nursing Sciences | 28 | Germany | Distress | Qualitative |
| Groves et al. 2022 | News reporting of suicide in nurses: A content analysis study | International Journal of Mental Health Nursing | 6 | United Kingdom | Completion | Quantitative |
| Groves et al. 2023 | Suicide, self-harm, and suicide ideation in nurses and midwives: A systematic review of prevalence, contributory factors, and interventions | Journal of Affective Disorders | 48 | United Kingdom | Distress/ ideation / completion | Review |
| Hofstetter and Mayer 2022 | Suicide Prevention: Protecting the Future of Nurses | American Journal of Nursing | 3 | United States | Intervention | Discussion pieces/commentaries/ editorials |
| Jahan et al. 2021 | COVID-19 suicide and its causative factors among the healthcare professionals: Case study evidence from press reports | Perspectives In Psychiatric Care | 99 | Bangladesh | Completion | Qualitative |
| James et al. 2023 | A deductive thematic analysis of nurses with job-related problems who completed suicide during the early COVID-19 pandemic: A preliminary report | Worldviews on Evidence-Based Nursing | 15 | United States | Completion | Qualitative |
| Kabir et al. 2023 | Association of workplace bullying and burnout with nurses' suicidal ideation in Bangladesh | Scientific Reports | 15 | Bangladesh | Ideation | Quantitative |
| Kelsey et al, 2021 | Suicidal Ideation and Attitudes Toward Help Seeking in U.S. Nurses Relative to the General Working Population | American Journal of Nursing | 69 | United States | Ideation | Quantitative |
| Lange 2023 | The hidden crisis of nurse suicide | Nursing | 6 | United States | Completion/ intervention | Discussion pieces/commentaries/ editorials |
| Lascelles et al. 2023 | Suicide among nurses: What can we do to protect our workforce? | Journal of Advanced Nursing | 5 | United Kingdom | Completion | Discussion pieces/commentaries/ editorials |
| Lee et al. 2023 | Deaths by Suicide Among Nurses A Rapid Response Call | Journal of Psychosocial Nursing And Mental Health Services | 41 | United States | Completion | Discussion pieces/commentaries/ editorials |
| Li et al. 2024 | Psychosocial characteristics pattern correlated with suicidal ideation and non-suicidal self-injury among nurse staff: a latent profile analysis | BMC Nursing | 3 | China | Ideation | Quantitative |
| Melnyk et al. 2024 | A study protocol for the modified interactive screening program plus MINDBODYSTRONG© RCT: A mental health resiliency intervention for nurses | Plos One | 3 | United States | Intervention | Intervention |
| Milner et al. 2016 | Suicide by health professionals: a retrospective mortality study in Australia, 2001-2012 |  | 168 | Australia | Completion | Quantitative |
| Mao et al. 2024 | Association between transition patterns of sleep problems and suicidal ideation in Chinese female nurses: A prospective study | Journal of Clinical Psychology | 7 | China | Ideation | Quantitative |
| NCISH 2020 | Suicide by female nurses: a brief report. 2020. | University of Manchester | 9 | United Kingdom | Completion | Quantitative |
| Petit et al. 2024 | Situational and Personal Predictors of Mental Health Outcomes Among Health Care Workers During Covid-19: Differences Between Nurses and Physicians | Psychiatria Danubina | 1 | Belgium | Distress | Quantitative |
| Petrie et al. 2023 | Suicide among health professionals in Australia: A retrospective mortality study of trends over the last two decades | Australian And New Zealand Journal of Psychiatry | 12 | Australia | Completion | Quantitative |
| Rees et al. 2024 | Interventions aimed at preventing suicide in the healthcare workforce: a systematic review. | Nursing Management | 1 | United Kingdom | Intervention | Review |
| Reid Ponte et al 2021 | An Interview with Judy Davidson: Nurse Suicide Risk Detection and Prevention | Journal of Nursing Administration | 0 | United States | Completion / intervention | Discussion pieces/commentaries/ editorials |
| Rebair et al. 2024 | Understanding the factors underpinning suicidal ideation amongst the UK nursing workforce from 2022 to 2024 | Royal College of Nursing | 0 | United Kingdom | Completion | Review |
| Silva et al. 2023 | SUICIDE ATTEMPT AT WORK, NURSING AND THE COVID-19 PANDEMIC | Revista De Gestao Social E Ambiental | 0 | Brazil | Ideation | Qualitative |
| Silva et al. 2015 | Depression and suicide risk among Nursing professionals: an integrative review | Revista Da Escola De Enfermagem Da Usp | 130 | Brazil | Ideation / completion | Review |
| Sofer 2021 | Female Nurses at Increased Risk for Suicide | The American Journal of Nursing | 0 | United States | Completion | Discussion pieces/commentaries/ editorials |
| Sofer 2018 | Suicide Among Nurses | American Journal of Nursing | 9 | United States | Completion | Discussion pieces/commentaries/ editorials |
| Stelnicki et al. 2020 | Suicidal Behaviors Among Nurses in Canada | Canadian Journal of Nursing Research | 42 |  | Ideation | Quantitative |
| Wang et al. 2024 | Associations between workplace violence and suicidal ideation among Chinese medical staff: a propensity score matching analysis | Psychology Health & Medicine | 2 | China | Ideation | Quantitative |
| Wei et al. 2023 | Insomnia, Suicidal Thoughts, and Mental Health Among Health-Care Workers During COVID-19 | Disaster Medicine and Public Health Preparedness | 2 | Taiwan | Ideation | Discussion pieces/commentaries/ editorials |
| Wells et al. 2024 | The Impact of Nurses’ Work Environment on Mental Health and Suicide | Critical Care Nurse | 3 | United States | Ideation/ completion | Discussion pieces/commentaries/ editorials |
| Whybrow et al. 2024 | The perceived helpfulness and acceptability of a bespoke psychological therapy service for registered nurses experiencing psychological distress: A qualitative study | Journal Of Advanced Nursing | 0 | United Kingdom | Intervention | Qualitative |
| Zeng 2018 | Chinese nurses are at high risk for suicide: A review of nurses suicide in China 2007-2016 | Archives Of Psychiatric Nursing | 49 | China | Completion | Qualitative |
| Zohn and Hovis 2024 | The impact of the global COVID-19 pandemic on risk factors for suicide in healthcare workers: A narrative review | Journal Of Clinical Nursing | 13 | United States | Completion | Review |
